# Cancer registries can provide evidence-based data to improve quality of care and prevent cancer deaths

**DOI:** 10.3332/ecancer.2014.413

**Published:** 2014-03-28

**Authors:** Christine Bouchardy, Elisabetta Rapiti, Simone Benhamou

**Affiliations:** Geneva Cancer Registry, Global Health Institute, University of Geneva, 55 Bd. de la Cluse, 1205 Geneva, Switzerland

**Keywords:** cancer registration, cancer control, evaluation, population-based, epidemiological research, quality of care

## Abstract

Today, many countries are increasing their efforts to ensure that all cancer patients receive the best possible care. Population-based cancer registries have adapted their registration to collect additional clinical variables to provide clinicians with unbiased population data on cancer treatment and survival. Taking several examples of epidemiological cancer research performed at the Geneva Cancer Registry, we aim to illustrate how cancer registries oversee the treatment and outcomes of cancer patients to help clinicians continually improve quality of care and prevent cancer deaths in the population.

## Introduction

War against cancer is unanimously accepted as a priority. About 14 million new cancers and eight million cancer deaths are occurring annually in the world and projections of cancer burden are alarming, with a predicted increase of 57 % in the next two decades [1].

Having continuous, robust, and unbiased population data on cancer occurrence is necessary to monitor the impact of the disease, to build public health priorities and to evaluate the efficacy of cancer control programs in the community. The primary aim of population-based cancer registries is to provide such data by collecting individual information on all patients diagnosed with cancer in the general population. The first population-based cancer registries were created in 1929 in Germany. Several hundred registries are today in activity worldwide covering about 21% of the world population [2]. Registration of data is standardised according to international guidelines to ensure quality, completeness, and comparability. Originally, the role of cancer registries was limited to describe the burden of the disease, trends, and geographical comparisons. Progressively, several cancer registries extended their data collection to provide survival data to assess the overall efficacy of the health care system. More recently, data registration was extended to clinical variables to respond to the growing need of information for the evaluation of generalisation of good practices, the disparities in care and long-term effects of treatment.

The population-based Geneva Cancer Registry, created in 1970, records all incident cancer cases occurring in the small population of the canton of Geneva (approximately 475,000 inhabitants). To perform relevant studies responding to clinicians’ and patients’ needs, the Registry has set up several multidisciplinary networks regrouping professionals of public and private sectors and patients.

## Objective

This article presents a selection of studies performed by the Geneva Cancer Registry providing epidemiological data for clinicians to improve the quality of patient care in the community.

## The Geneva Cancer Registry

The Cancer Registry extracts information from various sources and is considered accurate, as attested by its very low percentage of cases (<2%) recorded from death certificates only [3]. All hospitals, pathology laboratories, and private practitioners in the canton are requested to report every cancer case. Trained tumour registrars systematically extract data from medical and laboratory records. Physicians regularly receive enquiry forms to complete missing clinical data. Recorded data include socio-demographic informations (including country of birth, last occupation), health care sector, method of detection, type of confirmation of diagnosis, tumour characteristics (including stage of disease at diagnosis, treatments within the first six months (place, date, nature, and finality), second cancer occurrence, survival status, the exact cause of death, and place of death. For some cancer sites additional variables are recorded, such as family history of cancer, comorbidities, body mass index, recurrence, and so on. In 2011, more than 100,000 cancer patients were recorded in the Registry database.

## Examples of study

### 1. Impact of family history on cancer outcome

Breast cancer risk in Geneva is one of the highest in the world. Familial history of breast cancer is a well-established risk factor of breast cancer: a positive family history is one of the strongest predictors in a woman’s lifetime risk of developing breast cancer. *BRCA1 *and *BRCA2 *are the two major susceptibility genes involved in hereditary predisposition to breast cancer. In addition, carrying *BRCA1/BRCA2 *germline mutations has been associated with a high risk of contralateral breast cancer. Genetic counselling and testing is now routinely offered to individuals with increased probability of carrying *BRCA1/BRCA2* mutations.

Women with positive family history and clinicians have to face numerous decisions, including the choice of undergoing genetic counselling, testing for *BRCA1* and *BRCA2* mutations, screening, and prevention strategies, such as chemoprevention and prophylactic surgery.

Our team of cancer professionals, which include trained registrars, medical doctors, and statisticians, have setup the first population-based Familial Breast Cancer Registry in Switzerland with the financial support of the Swiss Cancer League. Since 1990, we have been collecting the family history for all breast cancer patients in the canton of Geneva. The Familial Breast Cancer Registry currently includes more than 7000 women diagnosed with invasive or *in situ *breast cancer since 1990. This collection of data provides an inestimable resource for breast cancer research.

To illustrate this topic, we chose two studies that were conducted to help clinicians managing women with positive family history of breast cancer [4, 5].

The first study aimed to evaluate for the first time how the risk of second breast cancer occurrence is affected by family history of breast cancer according to the tumour profile of the first and second breast cancers. A rather remarkable finding was the very strong risk of developing a second estrogen receptor (ER)-negative breast cancer among patients with strong family history of breast cancer. Patients with positive family history of breast cancer had a tenfold (standardised incidence ratio (SIR): 9.7, 95% CI: 3.6–21.1) higher risk of ER-negative second tumour which increased to nearly 50-fold (SIR: 46.2, 95% CI: 12.6–118.2) when the first tumour was also ER-negative. Our study also showed that the risk of second ER-negative breast cancer was particularly high for patients diagnosed during the last study period, i.e., when aromatase inhibitors treatment increased.

The second study concerns the determinants of use of genetic counseling among patients at high-familial risk, and the effects of genetic counseling on patients’ surveillance and outcomes. For this study, we linked the databases of the Oncogenetics and Cancer Prevention Unit at the Geneva University Hospitals and the population-based Geneva Cancer Registry. Overall, 11.2% breast cancer patients had genetic counseling; this proportion was 25.1% within the high familial risk group. Recent period of diagnosis, early onset breast cancer, tumour size, and chemotherapy treatment were statistically significantly associated with genetic counseling uptake in multivariate analysis. An increased risk of contralateral breast cancer of borderline significance was found for patients who had genetic counseling *versus *those who had not (adjusted hazard ratio [HR]: 2.2, 95% confidence intervals [CI]: 1.0–5.2, *P *= 0.063). Stratification by *BRCA1/BRCA2 *mutation status showed that the occurrence of contralateral breast cancer was eight fold higher among mutation carriers compared with non-carriers. Age-adjusted overall survival and breast cancer-specific survival were not significantly different between patients who underwent genetic counseling and those who did not.

These results were discussed with geneticists and patients from the breast cancer network. Two main issues were raised. The first was linked to the very high risk for women with positive family history to develop a second breast cancer, in particular an ER-negative tumour when the first breast cancer was also ER-negative. Our recommendations were that these women should receive an adequate surveillance of the controlateral breast and/or preventive surgical measures, as the use of anti-estrogen would probably have no effect on ER-negative tumours. The second concerned the low use of genetic counseling which could be either due to the lack of assessment of family history among patients with breast cancer or to a low propensity of physicians to refer high-familial risk patients to the genetic counseling. Improving the use of genetic counseling services for individuals at risk in a population level will permit to assess genetic risk, provide adequate surveillance, and an optimal management of these women and their families.

#### Importance and determinants of increased cancer mortality among patients of low social class

Due to early detection and progress in treatment, a remarkable improvement in survival is observed for many cancers in developed countries. However, social inequalities in cancer prognosis still remain, with patients of low socio-economic status having the poorest survival [6, 7]. Reasons at the origin of this over-mortality are far to be fully understood.

We therefore decided to assess the importance and determinants of inequalities in survival by socio-economic status using breast cancer as an example [8]. In Geneva, average income, life expectancy, and cancer survival are among the highest in the world; in addition, medical services are easily accessible and technical equipment are more than optimal [9]. We would expect social disparities to be minimal in our canton. We built a socio-economic indicator based on women’s last occupation and when absent on occupation of the spouse. Surprisingly, we observed a more than doubled breast cancer-specific mortality risk among patients of low socio-economic status as compared with those of high socio-economic status (unadjusted HR: 2.4, 95% CI: 1.6–3.5). As expected, women of low socio-economic status were more often foreigners, had less frequently screen-detected cancer, more advanced stage at diagnosis, and more frequently suboptimal treatments. This increased risk of breast cancer mortality was neither fully explained by patient and tumour characteristics nor treatment (adjusted HR: 1.7, 95% CI: 1.1–2.5). To better understand the reasons behind these results, we decided to collect additional information for a subgroup of these patients. We found that patients of low socio-economic status had longer delays between diagnosis and treatment, more often positive surgical margins, and less often access to new and more expensive treatments (manuscript in preparation).

Following these results, we suggested that the overall evaluation of the disease should include socio-economic status just as other classical prognostic factors. All women of low socio-economic status should benefit of support and surveillance to make sure that they have access to optimal treatment to prevent their inacceptable over-mortality.

### 2. Therapeutic progress is not always generalised in daily practice

At the end of the 1980s, randomised clinical trials provided evidence that adjuvant postoperative chemotherapy in colon carcinoma patients with regional lymph node metastasis (stage III colon cancer) increased the survival rates by approximately 30% [10–12]. Based on these results and given the uncommon toxic side effects of these treatments, therapeutic guidelines were established in 1990, recommending systematic adjuvant chemotherapy after surgery for stage III colon carcinoma [13].

To illustrate putative gaps between scientific knowledge and daily practice, we chose to describe the use of adjuvant therapy after surgery of stage III colon cancer using data from our Registry [14].

We were surprised to observe that only 30% of patients with stage III colon cancer received adjuvant therapy. The use of chemotherapy declined with increasing age at diagnosis but was not explained by patient’s refusal or by the presence of comorbidities. Particularly, low rates of use were also observed among foreigners and patients with low socio-economic status. After adjusting for patient’s selection bias, the risk of colon cancer death was significantly lower among patients who received chemotherapy than among those who did not (adjusted HR: 0.4, 95% CI: 0.2–0.7). The five-year survival found in our study for treated patients was very close to that observed in clinical trials [10, 15].

We concluded that adjuvant chemotherapy for stage III colon carcinoma patients had not reached its full potential in daily practice. The probability of being treated remained too low. We encouraged the medical community to change their practice to offer each patient the best chance of survival.

#### Long-term effects of anti-cancer treatment could kill after cure

With improving survival rates following cancer treatment, patients are increasingly likely to experience long-term adverse effects. Clinicians need to be aware of these effects. Several studies have reported that adjuvant radiation therapy for breast cancer is associated with an increased risk of cardiovascular mortality, probably due to the cardio toxicity of radiation [16]. This excess of cardiovascular mortality was especially observed in patients with left-sided breast cancer, where the heart is more exposed to ionizing irradiation. However, in these randomised trials, patients underwent radiation treatment before 1975, with techniques now considered as obsolete. Today, with more modern radiation techniques and generalisation of cardiac protection for left sided breast cancer, doses delivered to the heart are lower and the excess of cardiovascular mortality among patients with left breast cancer has decreased [17].

As cardiovascular diseases are the leading causes of death among women today, any increased risk of cardiovascular disease could have a high impact on mortality. We studied the risk of cardiovascular mortality occurring after radiation therapy among breast cancer survivors according to both laterality and breast cancer location [18]. We limited the study to lymph node-negative breast cancer patients for whom other putative cardio toxic adjuvant chemotherapy is usually not indicated. Overall, about 30% of deaths were due to cardiovascular mortality and 60% to breast cancer. Patients with inner-quadrant tumours had a more than doubled risk of cardiovascular mortality compared with patients with outer-quadrant tumours (adjusted HR: 2.5; 95% CI: 1.1–5.4). Patients with left-sided breast cancer had no more excess of cardiovascular mortality compared with patients with right-sided tumours.

We discussed these results with radio-oncologists and patients and concluded that the expected benefits of radiation therapy should be considered taking into account the risk of cardiovascular mortality. We advised to provide adequate cardiac protection when irradiating inner-quadrant breast cancer as it is done for left-sided breast cancer.

#### Observational studies can help clinicians when clinical trials are not available

Clinical trials are the best way to assess anticancer drugs effectiveness. However, such clinical trials are sometimes not feasible for ethical reasons or lack of patient recruitment. The best management of localised prostate cancer is still being debated because no randomised trials have established so far which of the four approaches, i.e., prostatectomy, radiotherapy, watchful waiting, and hormone therapy, offers the best chance of long-term survival [19]. Therefore, the choice of treatment is strongly influenced by patient and physician personal preferences and experience. Only observational studies considering patient’s selection bias can provide data to inform patients and physicians on mortality differences observed between the treatment groups.

We performed a study on long-term effects of treatment among men with localised prostate cancer in the context of the prostate cancer network using propensity score to correct for patient’s assignation in each group of treatment [20]. Treatments were very disparate in the population with 20% of prostate cancer patients receiving surgery, 24% radiotherapy, 45% watchful waiting, 9% hormonal therapy, and the others a mixture of different types of therapy. Treatment options only slightly influenced five-year prostate cancer-specific mortality, but had an important effect on long-term prostate mortality. At ten years, patients treated with radiotherapy or watchful waiting had a significantly increased risk of death from prostate cancer compared with patients who underwent prostatectomy (multiadjusted HR: 2.3, 95% CI: 1.2–4.3 and 2.0 95% CI, 1.1–3.8, respectively). The increased mortality associated with radiotherapy and watchful waiting was observed in patients younger than 70 years and in patients with poorly differentiated tumours. Patients who received hormone therapy alone had an increased risk of prostate cancer-specific mortality already at five years (HR: 3.5, 95% CI: 1.4–8.7]).

Our study results suggest that surgery offers the best chance of long-term prostate cancer-specific survival, in particular for younger patients and patients with poorly differentiated tumours.

We discussed the results of this study with clinicians and patients. We were all aware of the limitations of the study due to its observational nature and to the lack of records of adverse effects of treatment which are particularly severe for patients. However, several points emerged on the utility to propose curative treatments in particular for young patients with poorly differentiated tumour.

A second example of observational studies was the effect of omission of surgery among women with breast cancer. Once again clinical trials could not be performed for ethical reasons. We studied breast cancer women (aged less than 80 years) who refused surgery in daily practice [21]. We estimated the effects of patient’s refusal to undergo surgery on outcome after breast cancer and found a doubled risk of dying from breast cancer among patients who refused surgery as a curative treatment as compared with those who did not. This study should help clinicians to provide guidance to hesitant patients towards surgery with strong arguments.

#### Non-invasive treatment of precancerous lesions increases the risk of invasive cancer

Because treatment of cancer is often aggressive and accompanied by adverse effects, clinicians search for less invasive approaches, in particular for cancer precursors or *in situ* carcinomas. This is notably the case for cancer of the breast, skin, and cervix.

Treatment of high-grade CIN II and CIN III includes surgical excision or other destructive techniques. Several studies demonstrated that women treated for CIN II/CIN III had a higher risk of developing cervical cancer than the general population [22].

We present here a study, which aimed to investigate the association between treatment modalities for CIN III and *in situ *cervix carcinoma and the risk of subsequent invasive cervix cancer [23]. Overall, the risk of invasive cervix cancer was much higher for those patients than in the general population (SIR: 5.1, 95% CI: 3.0–8.1). Compared with patients with excisional treatment, the risk was about tenfold increased for women who had other treatment approaches (multiadjusted HR of invasive cervical cancer: 9.4, 95% CI: 2.8–32.2), such as cryotherapy, laser vaporisation, electro-cautery, diathermy, and cold coagulation.

After discussions with the clinicians about these results, we came to the conclusion that excisional treatment should be recommended for precancerous cervical lesions.

#### Undertreatment among elderly is often not justified and increases cancer deaths

The increasing life expectancy in developed countries and the growing incidence rates of cancer with increasing life ineluctably result in an important increase of older cancer patients. Old patients were until recently excluded from clinical trials [24]. Treatments of older patients remain influenced by uncertainty concerning the natural history of the disease with the general belief that cancer among elderly grows more slowly and is less aggressive than among younger patients. In addition, elderly individuals are thought to support less anticancer treatments due to the general deterioration of their health or to be less compliant because of their age and social isolation. Finally, the presence of other comorbidities will lead to treatment interactions and cancer deaths will be out weighted by deaths from other causes. Therapies for elderly patients depend on the physician’s and patient’s choices and beliefs, and are usually less aggressive.

We investigated treatment and prognosis among elderly breast cancer patients (≥80 years old) to assess putative undertreatment and its effect on patient’s prognosis [25]. Only, 1/4 of women presented with stage I breast cancer. Assessment of tumour characteristics was particularly low: 15% of patients had no microscopic histology and 20% were diagnosed by cytology only. A high proportion of breast cancers was reported as carcinoma not otherwise specified (23%) with an important proportion of unknown grade (49%) or unknown estrogen receptor status (74%). Elderly women with adequate tumour assessment did not have more indolent breast cancer than younger postmenopausal women. We observed great disparities in the treatments received. Overall, 12% of women had no treatment, 32% received tamoxifen only, 7% had breast-conserving surgery only, 33% had mastectomy, 14% had breast-conserving surgery plus adjuvant therapy, and 2% received miscellaneous treatments. Elderly women with poor general health status were less likely to undergo surgery. However, comorbidities were present in only 17% of the women who did not have surgery. Among elderly women who had surgery, 7% had positive surgical margins and 62% axillary lymph node investigation. Tamoxifen was given regardless of the results of the estrogen receptor status testing: the proportion of women receiving tamoxifen was 61%, 60%, and 61% for positive, negative, and unknown estrogen receptor status, respectively.

Five-year specific breast cancer survival was 46%, 51%, 82%, and 90% for women with no treatment, tamoxifen alone, mastectomy, and breast-conserving surgery plus adjuvant treatment, respectively. Compared with the non-treated group, the adjusted HR of breast cancer mortality was 0.4 (95% CI: 0.2–0.7) for tamoxifen alone, 0.4 (95% CI: 0.1–1.4) for breast-conserving surgery alone, 0.2 (95% CI: 0.1–0.7) for mastectomy, and 0.1 (95% CI: 0.03–0.4) for breast-conserving surgery plus adjuvant treatment.

We concluded that elderly women have a fairly poor diagnosis investigation, delayed diagnosis, do not have more indolent breast cancers than younger women and are subject to great treatment disparities. Half of them had suboptimal treatment with high mortality risk from breast cancer as consequence. This suboptimal approach is only very partly explained by presence of comorbidities or patient’s refusal. These results were presented to clinicians together with data on life expectancy, which remains high (nine years) in Switzerland for women alive at the age of 80 years. We formulated and disseminated the following recommendations: elderly women must benefit from earlier diagnosis and better diagnostic characterisation; treatment needs to be adapted to the patient’s general health status and comorbidity conditions, and should offer the best chances of survival; elderly women must be informed about available treatment options and the consequences of undertreatment to avoid unacceptable excess of breast cancer mortality.

### 3. Evaluate the effect of surgeon’s experience on quality of care and cancer prognosis

The present study ‘Breast cancer quality of care and outcome according to surgeon’s caseload’ has received the Swiss Bridge Award 2013. In most countries, an accreditation scheme for breast services has been recommended to provide the best practice standards and guidelines for breast cancer treatment. In Switzerland, a large number of practitioners in the public and private sectors provide care for breast cancer patients. In some urban areas, more than 50% of breast cancer cases are treated in the private sector, a proportion probably higher than in most European countries. One of the most important criteria for accreditation of breast cancer units is to treat at least 150 new cases per year. However, no study in Switzerland has assessed the effects of surgeon’s caseload on both breast cancer quality of care and prognosis. This is the aim of this study.

## Conclusion

With this selection of studies, we aimed to give some examples of how population-based cancer registries can directly improve patient care by involving the clinicians all along the research continuum from the definition of the priorities to the proposition of actions as to improve the quality of care. This approach could be reinforced by creating multidisciplinary research networks in which both clinicians and patients are involved as key partners.

## The future

The Geneva Cancer Registry has set new challenges for the 21st century: improve the etiologic research to identify the causes of cancer; compare the cost-benefits of different treatments/diagnostic procedures; include quality of life in the evaluation of patient care.

Cancer registry epidemiologists should closely work with professionals from other cancer specialties in the context of multidisciplinary research networks. This approach will help cancer registries to orientate their research on clinicians and patient’s actual needs and discuss within the network the clinical implication of these research results.

The information coming from the genome wild association studies should be considered because the interaction between genetic and environmental factors is probably the key for understanding not only carcinogenesis but also drug effectiveness/resistance or side effects occurrence. Therefore, cancer registries should play a role in collecting data of cancer patient’s genetic profiles and/or linking with available biobanks.

In addition, the potential public health use of `big data’ extends well beyond genomics. Registries should have access to available data on risk factors including environmental exposures, personal habits, familial links as well as treatment details like palliative care, costs, and quality of life. We need new technologies to facilitate the integration of this information.

New ethical problems and data protection requirements will emerge. The realisation of all these projects could be hampered if the new European Union Privacy Protection bill and the pending Swiss law on cancer registration require an explicit informed consent to use health data for public health monitoring and research [26]. Cancer registries will continue their important activities, adapting to medical progress and future challenges in cancer research.
